# TiO_2_–ZnO functionalized low-cost ceramic membranes from coal fly ash for the removal of tetracycline from water under visible light

**DOI:** 10.1186/s11671-024-04178-3

**Published:** 2025-01-03

**Authors:** Lawrence Sawunyama, Opeyemi A. Oyewo, Seshibe S. Makgato, Mokgadi F. Bopape, Damian C. Onwudiwe

**Affiliations:** 1 Materials Science Innovation and Modelling (MaSIM) Research Focus Area, Faculty of Natural and Agricultural Sciences, North-West University, Mafikeng Campus, Private Bag X2046, Mmabatho, 2735 South Africa; 2 Department of Chemistry, School of Physical and Chemical Sciences, Faculty of Natural and Agricultural Sciences, North-West University, Mafikeng Campus, Private Bag X2046, Mmabatho, 2735 South Africa; 3 Department of Chemical & Materials Engineering, College of Science, Engineering and Technology, University of South Africa, Johannesburg, South Africa; 4 Department of Chemical, Metallurgical and Material Engineering, Tshwane University of Technology, Private Bag x680, Pretoria, 0001 South Africa

**Keywords:** Photocatalytic ceramic membranes, Coal fly ash, Heterojunction photocatalyst, Tetracycline, Ultrafiltration

## Abstract

Hybrid wastewater treatment systems offer viable solutions to enhance the removal of complicated contaminants from aqueous system. This innovation has opened new avenues for advanced wastewater treatment processes. Herein, a novel TiO_2_–ZnO functionalized coal fly ash-based ceramic membrane was fabricated by utilizing a combined pressing and sintering method. The intrinsic properties of the functionalized membranes were characterized and their chemical and physical properties such as chemical stability, mechanical stability, water absorption, and porosity were established. The shape, crystallinity, thermal characteristics, and functional groups present were also determined using SEM, XRD, TGA, and FTIR studies, respectively. The results showed that the ceramic membrane functionalized with 0.5 g of TiO_2_–ZnO and sintered at 850 °C exhibited the best thermal, and chemical stability, and possessed the required porosity for ultrafiltration applications. Photocatalytic degradation of tetracycline (TC) as a model pollutant was examined and the optimum efficiency of 77% was achieved within 100 min of visible irradiation using the functionalized membrane. Moreso, the functionalized membrane was found to be stable with 73% degradation efficiency after 5 consecutive cycles of reusability study, showing negligible loss of efficiency. The scale-up of photocatalytic ceramic membranes and their utilization in real industrial applications will confirm their robustness.

**Graphical Abstract:**

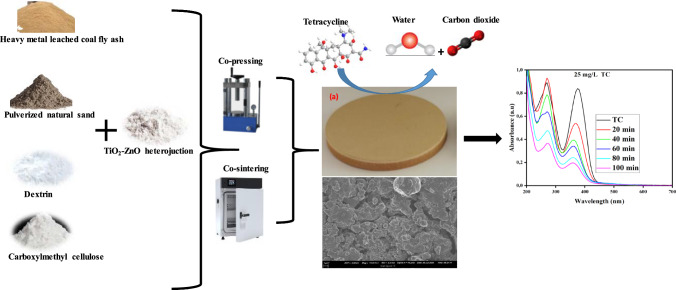

## Introduction

The use of ceramic membranes in filtration technology for wastewater treatment applications has evolved rapidly on a global scale due to its excellent filtration effectiveness, longer service life, lower susceptibility to fouling, and chemical and thermal stability [1, 2]. However, high energy requirements for sintering, high operational cost and limited performance in the mineralization of contaminants are identified as major drawbacks in real industrial utilization [3–5]. This has, thus, directed research to focus on the fabrication of ceramic membranes using inexpensive and easily accessible materials. For example, studies revealed that clay, bentonite, kaolin, sand, and other materials were successfully used for the fabrication of ceramic membranes [6–9]. These materials contain substantial amounts of SiO_2_, CaO, and Al_2_O_3_, making them an ideal precursor for ceramic membrane fabrication [10]. In our previous study, fabrication of a ceramic membrane from coal fly ash and natural sand at a sintering temperature that was lower than that of commercial starting materials was reported. The membrane demonstrated good water absorption capabilities in addition to good thermal and chemical durability [11].

To overcome the inefficiency of ceramic membranes in degrading contaminants in wastewater, the functionalization of ceramic membranes with photocatalysts has recently been explored. This hybrid method is based on exploring the combined advantages of photocatalysis and ceramic membrane technologies in order to maximize their benefits and minimize their drawbacks, hence reducing adverse effects on the economy and environment [12]. Metal oxide semiconductors are the major photocatalysts used to functionalize ceramic membranes [13], due to the generation of reactive species such as superoxide and hydroxyl radicals, which possess strong oxidizing capabilities and can effectively degrade organic pollutants [14–16]. Metal oxides such as ZnO and TiO₂ are widely regarded as safe and environmentally friendly materials. These give them advantage over advanced materials such as MXenes, Layered Double Hydroxides and covalent organic frameworks (COFs), whose environmental implications of synthesis procedure are still under investigation. These advanced materials often involve more complex synthesis and handling processes, which can hinder their scalability and practical applications [17, 18]. Another key advantage of metal oxide photocatalysts is their ease of fabrication compared to other photocatalytic options. This simplicity makes them a viable choice for large-scale applications. Additionally, their straightforward production facilitates their integration into existing technologies, such as coatings and composite materials. However, the limited absorption of solar light, poor adsorption characteristics, and high recombination rates impede photocatalytic activity of these metal oxide semiconductors. Consequently, semiconductor hybridization to form heterojunctions and elemental doping have been extensively and effectively studied to meet the necessary properties for industrial applicability [19, 20] [21, 22]. However, only relatively few studies exist on the removal of contaminants such as pharmaceuticals from wastewater by utilizing metal oxide functionalized ceramic membranes. For example, Li et al. [23] reported the degradation of diclofenac, naproxen, and carbamazepine utilizing graphene oxide-titanium dioxide photocatalytic ceramic membranes. In a similar study, Ghaderi, Lahafchi [24] successfully decomposed diphenhydramine (DPH) with a CuO-TiO_2_ functionalized ceramic membrane. Studies involving antibiotics degradation by employing a functionalized ceramic membrane is not well explored, hence the focus of this study.

Antibiotics are a class of pharmaceutical products that are widely used in aquaculture, agriculture, and human and animal therapy due to their effectiveness and affordable price [25]. However, excessive use of these antibiotics combined with improper environmental discharge can lead to very serious consequences for the environment. Tetracycline as a model antibiotic, which could cause harm to humans’, microorganisms’, and animals’ health when misused [26, 27]. Trace levels of tetracycline in drinking water can lead to significant adverse effects on humans’ health and aquatic lives over extended periods. In humans, exposure can result in permanent discoloration of teeth in foetuses and children under eight years old, as well as impairments in foetal long bone growth [28]. In plants, tetracycline has been shown to restrict growth, affect oxidative responses, and alter phytochemical composition [29]. In terrestrial and aquatic systems, it significantly impacts growth and reproduction [30]. With tetracycline consumption projected to increase by 30% by 2030 [31], and given that it is the second most consumed pharmaceutical drug, its residues in wastewater are not effectively removed by conventional treatment methods employed in most wastewater treatment plants. The challenge of removing tetracycline from wastewater is compounded by its coexistence with other pharmaceutical contaminants, heavy metals, and organic pollutants, such as dyes. The presence of electron-donor groups in tetracycline allows it to form stable compounds with various ions, making removal more difficult [32]. Additionally, its complex chemical structures and their low concentrations in wastewater also make it difficult to remove it from wastewater [33]. This highlights the urgent need for the development of cost-effective and efficient wastewater treatment techniques capable of degrading tetracycline.

Therefore, this study focuses on the fabrication of novel sustainable photocatalytic ceramic membranes using TiO_2_–ZnO heterojunction, natural sand, and coal fly ash for the removal of tetracycline. The photocatalytic ceramic membrane was fabricated through the co-pressing and co-sintering methods. The operating parameters including pH, tetracycline concentration, pressure and catalyst concentration were also investigated to evaluate the filtration and photocatalytic processes of the functionalized ceramic membrane on the removal of tetracycline. The feasibility studies on the industrial applicability of this synthesized functionalized membrane were also evaluated.

## Experimental

### Materials

TiO_2_–ZnO heterojunction was prepared using zinc acetate [Zn(CH_3_COO)_2_⋅2H_2_O, ≥ 98%], titanium tetra-isopropoxide [Ti(OCH(CH_3_)_2_)_4_, 98%], acetic acid [C_2_H_4_O_2_, > 99%], and sodium hydroxide [NaOH, 98%], that were procured from Merck, South Africa. The primary starting materials for ceramic membrane production were coal fly ash (CFA) and natural sand (NS) from national power utility in Zimbabwe. Carboxymethylcellulose sodium salt and dextrin (C_6_H_12_O_6_)x were acquired from Merck in South Africa and used as additions to bind raw ingredients and produce pores respectively. Merck, South Africa supplied tetracycline (TC), which served as the pharmacological model for the photocatalytic degradation test.

### Synthesis of TiO_2_–ZnO heterojunction

TiO_2_–ZnO heterojunction was prepared according to our previous work [20] with some modifications. The heterojunction photocatalyst was synthesized using titanium tetra-isopropoxide and zinc acetate solutions. Firstly, ZnO sol was prepared by dissolving pre-weighed zinc acetate in 100 mL of distilled water. About 100 mL of 0.5 M NaOH was added to the mixture under vigorous stirring for 2 h. TiO_2_ sol was prepared by adding (in drops) the measured volume of titanium tetra-isopropoxide into 100 mL of distilled water with continuous stirring for 30 min at 80 ℃. Then, 50 mL of ethanol was added in drops to the white precipitate solution. The resultant mixed solution was further stirred for 2 h at 25 °C. After 24 h of continuous stirring with a magnetic stirrer, the produced ZnO and TiO_2_ sol were transformed into a gel-like solution. The resultant TiO_2_–ZnO gel was centrifuged and rinsed with distilled water, followed by drying at 120 °C for 8 h. Thereafter, the crumps were crushed into a fine powder with a pestle and mortar before calcining for 2 h at 850 °C in a muffle furnace.

### Thermal treatment of coal fly ash and leaching of heavy metals from coal fly ash

Pre-weighed coal fly ash was thermally treated in a muffle furnace set at 1000 °C for 1 h to remove any unburnt carbon and volatile matters. The leaching experiments were conducted according to the procedure reported in our previous work [11]. Specifically, 1 g of thermally treated coal fly ash was added to 50 mL of 1 mol/L acetic acid solution and stirred continuously at a moderate speed for 6 h. Following this, the mixture was allowed to stand at room temperature for 24 h and then filtered. The resulting residue was washed with distilled water to eliminate any residual acetic acid and subsequently dried at 110 °C for 10 h.

### Preparation of TiO_2_–ZnO functionalized CFA/NS ceramic membrane

**Fig. 1:**
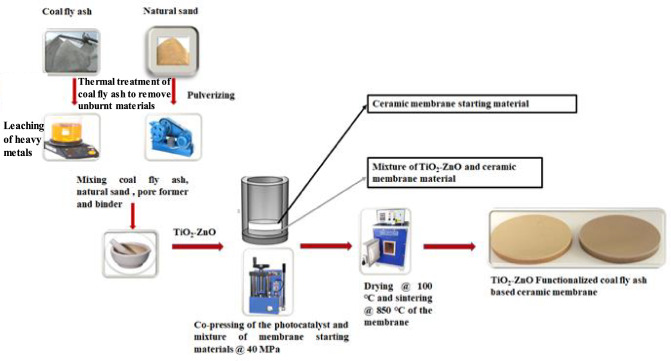
Schematic diagram of the fabrication process of TiO_2_–ZnO functionalized coal fly ash-based ceramic membranes by the co-pressing and co-sintering technique

The compositional ratios of the starting materials used in the fabrication of pristine coal fly ash-based ceramic membranes (PM) and functionalized coal fly ash based ceramic membranes (PCM) A, B and C are presented in Table 1. The photocatalytic ceramic membranes were fabricated using the co-pressing and co-sintering method, which has not been extensively used in fabricating functionalized ceramic membranes (Fig. 1). About 1 g of the precursor material was mixed with a pre-weighed, un-calcinated TiO_2_–ZnO photocatalyst, and the mixture was pulverized. The photoactive layer was formed by placing the grounded mixture on the mold's base, and the supporting layer was formed by adding pre-weighed ceramic membrane precursor material to the mold. The resultant mixture was uniaxially pressed for 3 min at a pressure of 40 MPa, resulting in a flat ceramic membrane with a defined diameter of 40 mm. The fabricated photocatalytic ceramic membranes were dried in a hot air oven at 110 °C for 10 h for complete removal of loose moisture. To avoid thermal shocks and cracks, the membrane was then sintered in a furnace at a rate of 5 °C min^−1^ up to 850 ℃ and maintained for 2 h. Thereafter, the membranes were cleaned in an aqueous ultrasonic bath to eliminate of any loose particles that had stuck to the surface of the membrane.

**Table 1:** The compositional ratios of the Materials used to fabricate the photocatalytic ceramic membranes

Raw materials	Weight of different raw materials used for various fabricated membranes			
	PM	PCM A	PCM B	PCM C
Ceramic membrane precursor material (g) (Mixture of coal fly ash (70 g), natural sand (20 g), dextrin (3 g) and sodium carboxymethyl cellulose (7 g))	9	8.75	8.50	8.25
TiO_2_–ZnO (g)	–	0.25	0.50	0.75

### Characterization of the photocatalytic ceramic membranes

Quanta FEG 250 Environmental scanning electron microscope (ESEM) coupled with EDX was utilized to characterize the morphology and elemental composition of the fabricated membranes. TG-DTA (TG/DTA 6300, Seiko Co., Ltd.) was used to evaluate the thermal stability of the ceramic membrane. X-ray diffraction (Bruker D8 Advance X-ray diffractometer (Karlsruhe, Germany) was used to determine the phase composition of the membranes. Fourier transform infrared spectroscopy (Bruker alpha-P FTIR spectrophotometer) was used to determine the functional groups present in the membrane and the photocatalyst. The three-point bending load method was used to assess the mechanical strength of the ceramic membranes.

### Water absorption, porosity and pore size

The water absorption and porosity of the fabricated functionalized ceramic membranes were determined using the gravimetric method. First, the ceramic membranes were weighed to obtain their dry weight (***dw***). The membranes were then submerged in distilled water at room temperature for 24 h. After this process, they were removed, wiped to remove excess water, and reweighed to obtain the wet weight (***ww***). Water absorption and porosity were calculated using the Archimedes principle. Average membrane pore size was determined using the filtration velocity method and calculated using the Guerout–Elford–Ferry Eq. 3.

1 $$Water\, absorption \left( \% \right) = \frac{ww - dw}{{ww}}*100$$

2 $$Membrane\, porosity \left( \varepsilon \right) = \frac{ww - dw}{{A*\rho *{\text{L}}}}*100$$

3 $$Membrane\, mean\, pore\, radius (r_{m) = } \sqrt {\frac{{2.9 - 1.7\varepsilon *8\eta {\text{L}}Q}}{\varepsilon *\Delta P*A}}$$

where ***ww*** = wet weight of the membrane sample (g), ***dw*** = dry weight of the membrane sample (g), ***A*** = the fabricated membrane’s effective surface membrane area (m^2^), **ρ**_**=**is_ the density of water **(kg/m**^**3**^**), L = **membrane thickness **(m),** rm = mean pore radius (µm), ***η*** = water viscosity (8.9 × 10^−4^ Pa s), ***Q*** = volume of permeate water (m^3^/s), and ***∆P*** = is operational pressure (Pa).

### Chemical and mechanical stabilities

**Fig. 2:**
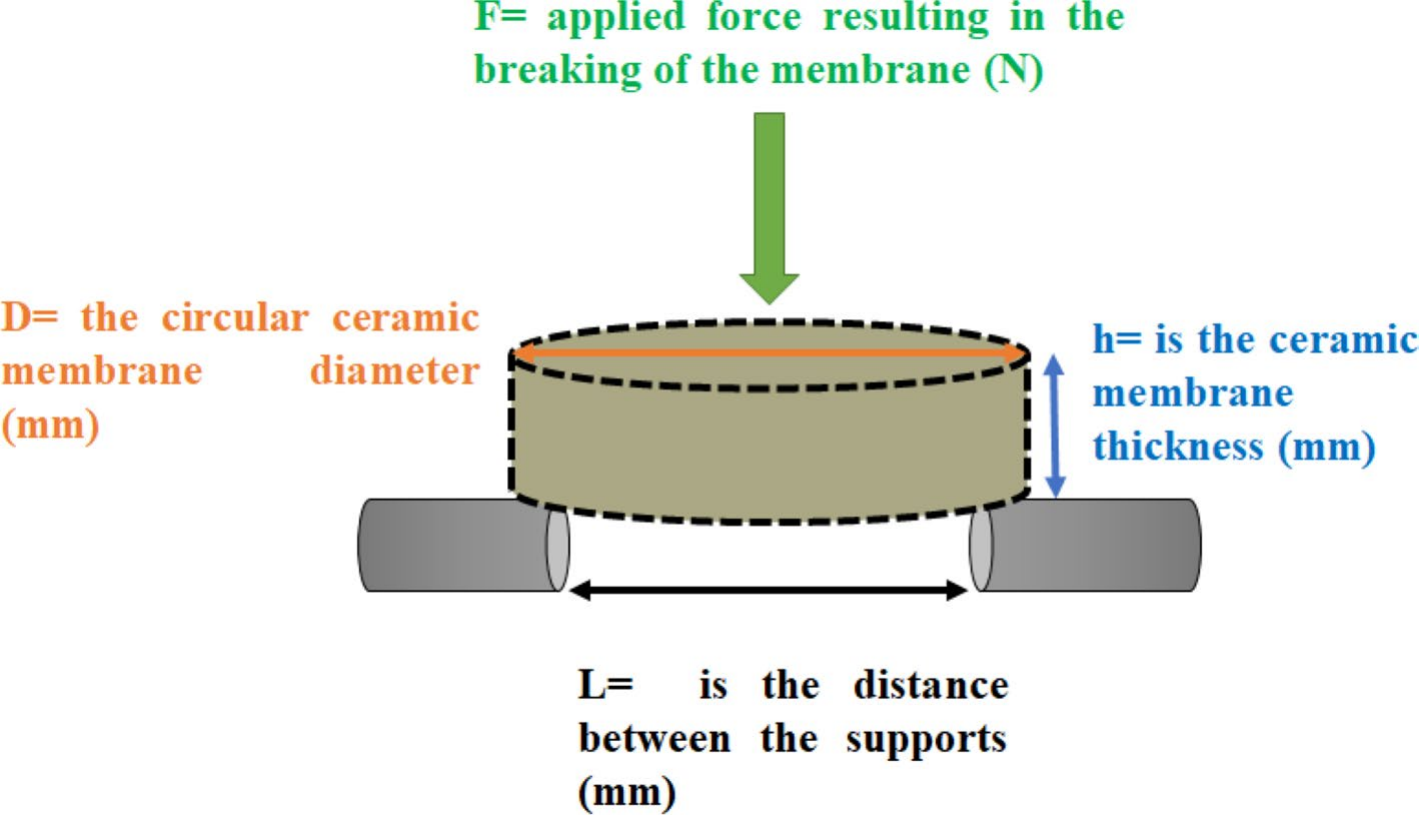
Schematic representation of the three-point bending test

The chemical stability of the fabricated ceramic membrane was evaluated using hydrochloric acid and sodium hydroxide at pH of 1.5 and 13 respectively [34]. First, the ceramic membranes were weighed to obtain their initial weight (***iw***). They were then submerged in a test medium (HCI and NaOH) at room temperature for 1 week. After this period, they were removed, wiped to remove excess test medium, and dried at 100 °C for 12 h, and then reweighed to obtain the final weight (***fw***). The chemical resistance of the ceramic membrane was evaluated in terms of weight loss using Eq. 4. The mechanical strength of the fabricated functionalized ceramic membranes was evaluated using a three-point bending test, as shown in Fig. 2. The flexural strength (MPa) of the circular flat ceramic membrane was calculated using Eq. 5.

4 $$Chemical\, stability \left( \% \right) = \frac{iw - fw}{{iw}}*100$$

5 $$Flexural\, strength \left( {MPa} \right) = \frac{3FL}{{2Dh^{2} }}$$

### Pure water permeability and tetracycline removal by filtration studies

A laboratory dead-end filtration setup was employed to evaluate the pure water permeation and tetracycline removal efficiency of the fabricated membranes. The filtration process was driven by pressure generated from nitrogen gas. Pure water flux (L/m^2^/h) and water permeability (L/m^2^/h/kPa) were calculated using Eqs. 6 and 7 respectively. The removal efficiency of tetracycline by both the pristine and functionalized ceramic membranes was assessed under uniform pressure conditions. The permeate was subsequently analyzed using a UV spectrophotometer, and the percent rejection (%R) was calculated using Eq. 8.

6 $$Pure\, water\, flux \left( J \right) = \frac{V}{A\Delta T}$$

7 $$Water\, permeability (J^{1} ) = \frac{V}{A\Delta TP}$$

where*** V*** is the volume of permeate in (L), ***A*** is the fabricated membrane’s effective surface membrane area (m^2^), ***ΔT*** is the time taken to collect the permeate (h), and ***P*** is the transmembrane pressure (kPa).

8 $$R\left( \% \right) = \frac{{C_{0} - C_{t} }}{{C_{0} }}*100$$

where ***C***_***0***_ and ***C***_***t***_ are the initial concentrations of Tetracycline and the concentration at time *t*, respectively.

### Procedure for photocatalytic degradation of tetracycline

Evaluation of the photocatalytic degradation of tetracycline by TiO_2_–ZnO functionalized coal fly ash-based ceramic membrane was performed in a photocatalytic reactor system by irradiating with light source from a 300 W of Xe lamp. To attain equilibrium before the reaction, the fabricated ceramic membrane was immersed in a solution of tetracycline and kept in the dark for 20 min. The suspension was constantly agitated while exposed to light. Analyte solutions were collected in 5 mL aliquots at 20-min intervals, instantly filtered through a microporous membrane, and the concentration of the permeate solutions was evaluated using a UV–vis spectrophotometer in the range of 200–900 nm wavelength. Equation 9 was used to calculate the percentage of tetracycline eliminated.

9 $$Degradation\, efficiency \left( \% \right) = \left( {1 - \frac{{C_{i} }}{{C_{f} }}} \right)*100$$

where ***C***_***i***_ and ***C***_***f***_ are tetracycline concentrations before and after photocatalysis respectively.

## Results and discussion

### Physical observations

**Fig. 3:**
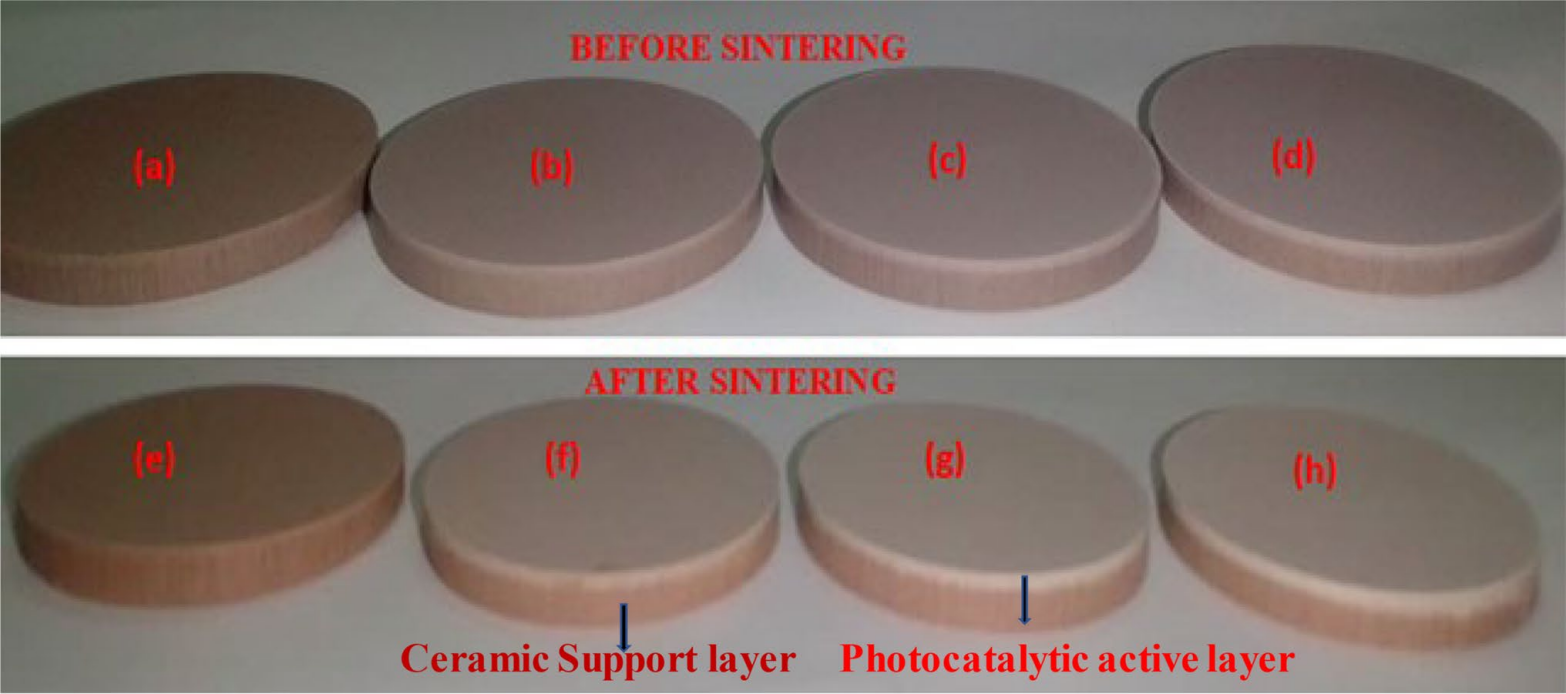
Pristine and functionalized ceramic membrane before and after sintering. Pristine coal fly ash-based ceramic membrane (**a** & **e**) and TiO_2_–ZnO functionalized coal fly ash-based ceramic membranes at different photocatalyst loading of (**b** & **f**) 0.25 g, (**c** & **g**) 0.50 g, and (**d** & **h**) 0.75 g

Figure 3 shows a picture of pristine coal fly ash-based ceramic membrane (a & e) and TiO_2_–ZnO functionalized coal fly ash-based ceramic membranes at different photocatalyst loading of (b & f) 0.25 g, (c & g) 0.50 g, and (d & h) 0.75 g. A slight but observable structural difference exist between the pristine and functionalized ceramic membranes. The pristine membrane is expected to exhibit a single-layered structure, whereas the functionalized membranes display a two-layered structure. The thickness of the surface-active layer of the functionalized ceramic membranes also increased proportionally with higher photocatalyst loading as shown in Table 2. The surface of the photocatalyst-functionalized ceramic membrane may be less coarse compared to the pristine membranes. This is due to the TiO_2_–ZnO incorporating into the matrices and chemically interacting with the ceramic membrane material, hence contributed to a more uniform surface.

**Table 2:** A summary of pristine and functionalized ceramic membrane properties

Membrane code	Thickness of surface active layer (mm)	Thickness of ceramic support layer (mm)	Water absorption (%)	Open porosity (%)	Crystallite size (nm)	Pore diameter (nm)	Water-Flux (L/m^2^/h)
PM	–	3.90	24.16	39.45	30.68	720	40.6
PCM 0.25	0.02	3.88	22.09	36.20	33.17	169	18.6
PCM 0.50	0.04	3.86	21.96	34.94	36.17	140	14.2
PCM 0.75	0.06	3.84	21.32	34.26	37.60	108	9.3

### Water absorption, porosity and pore size

The porosity and water absorption characteristics of the fabricated ceramic membranes were evaluated using Archimedes’ principle with water as the wetting liquid. As shown in Table 2, pristine coal fly ash-based ceramic membranes exhibited higher water absorption and porosity levels as compared to the functionalized ceramic membranes. As the loading of the TiO_2_–ZnO photocatalyst increased on the functionalized membranes, both porosity and water absorption decreased. This trend could be attributed to the increased occupation of available voids by the photocatalyst, thereby reducing the overall pore radius of the functionalized ceramic membranes [35]. The functionalization of the ceramic membranes can alter the membrane's selectivity and permeability, thereby impacting its filtration efficiency or separation applications [36]. Conversely, functionalizing ceramic membranes with nanoscale materials can facilitate the production of membranes with smoother surfaces. This has the potential to mitigate fouling and enhances the cleaning ability of the membrane, thereby improves the overall membrane performance. A study conducted by Alias et al., [37], revealed a consistent findings regarding the influence of functionalizing ceramic membranes on pore radius, porosity, water absorption, and density. Table 2 also shows the water flux of pristine coal fly ash-based ceramic membrane and TiO_2_–ZnO at different catalyst loading of 0.25, 0.50 and 0.75 g. The water flux of these fabricated membranes evaluated at the transmembrane pressure of 100 kPa followed the order of PM > PCM 0.25 > PCM 0.50 > PCM 0.75. The decrease in water flux occurred as the catalyst loading increased from 0.25 g to 0.75 g, and could be attributed to the significant reduction in pore size. This might be caused by the deposition of nanoparticles which occupied the membrane pores, thereby reduced their effective diameter and the membrane's overall permeability. This blockage also reduces the available space for contaminants to interact with the catalyst. Furthermore, a higher concentration of the catalyst could cause an increase in aggregation, lowering pore diameters and impeding water flow [38]. The reduction in porosity has a more substantial impact on water flux than the expected increase in hydrophilicity from the functionalization of the ceramic membrane with TiO_2_–ZnO nanoparticles. Marzouk et al. [39], reported similar findings, highlighting the influence of photocatalyst concentration on the water flux of functionalized ceramic membranes.

### Chemical and mechanical properties

**Fig. 4:**
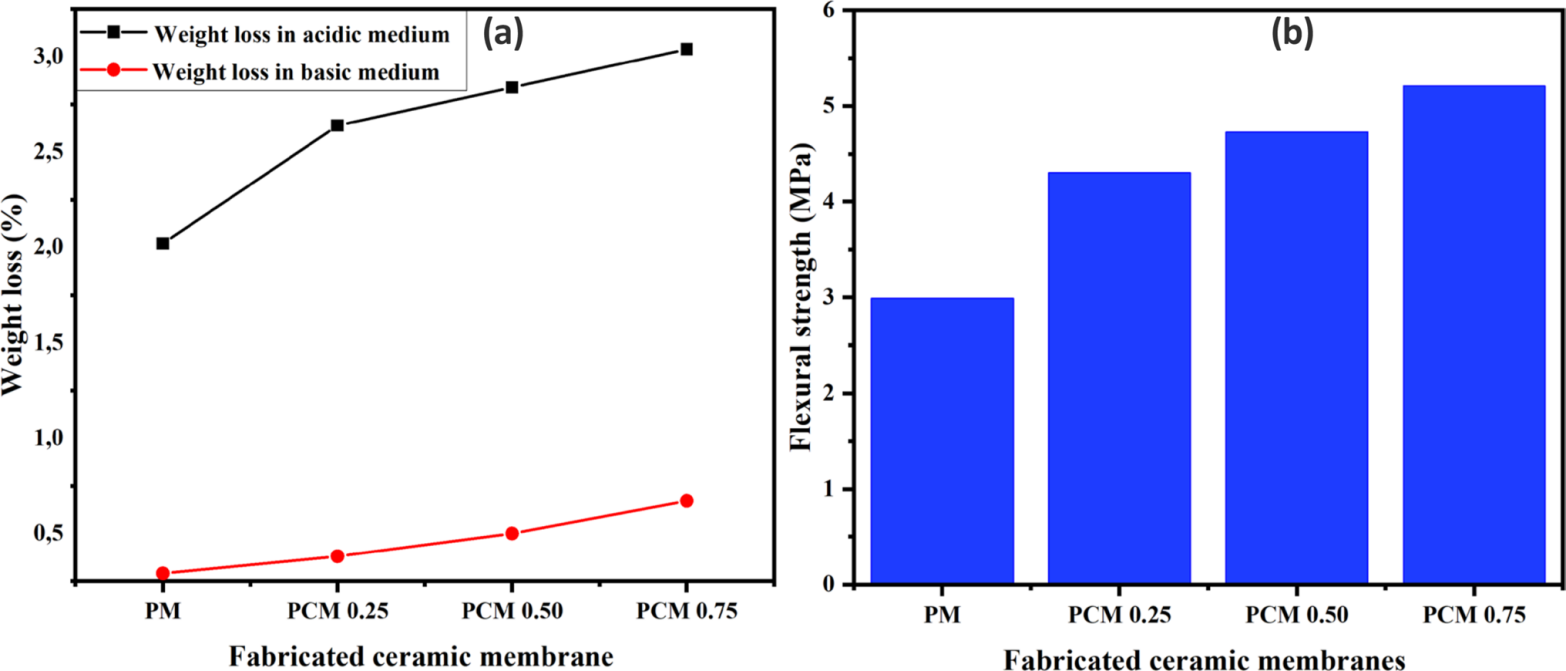
(**a**) Chemical stability and (**b**) Flexural strength of coal fly ash-based ceramic membrane (PM) and functionalized ceramic membranes at different photocatalyst loading of 0.25 g (PCM 0.25), 0.50 g (PCM 0.50) and 0.75 g (PCM 0.75) sintered at 850 ℃ for 2 h

The result of the acid–base stability of the fabricated ceramic membranes is shown in Fig. 4a. Both the pristine and functionalized coal fly ash-based ceramic membranes exhibited high chemical stability in both test media. This could be attributed to the high sintering temperature that promotes the shrinking of grains and decomposition of acid and base leachable compounds. It is also related to its ability to drive off impurities or residual organic compounds that are present in the ceramic material [40, 41]. The lower porosity limits the pathways through which chemicals can penetrate and react with the ceramic matrices. Results in Fig. 4a also show that the stability towards the acidic condition is lower as compared to the basic condition. Functionalized coal fly ash-based ceramic membranes showed higher weight loss in acidic environments compared to pristine ceramic membranes due to the presence of ZnO, MgO, CaO, and Na_2_O which are reactive with concentrated acids [42]. Figure 4b shows that pristine coal fly ash-based ceramic membranes had lower mechanical strength compared to functionalized ceramic membranes. Flexural strength increased from 4.30 MPa to 5.21 MPa when the number of TiO_2_–ZnO nanoparticles loaded increased from 0.25 g to 0.75 g. The observed trend could be attributed to the rise in crosslinking points between the uniformly distributed smaller TiO_2_–ZnO photocatalyst particles and the ceramic matrices [43]. The enhancement in crosslinking resulted to an increase in the rigidity of the surface of the functionalized ceramic membrane. The increase in mechanical strength could also be ascribed to the reduction of voids within the functionalized membranes, which led to a denser and more cohesive structure. However, there are not much difference between the mechanical strength of the pristine and the functionalized ceramic membranes. This could be due to their supporting layer being made from the same material composition.

### Fourier transform infrared spectra

**Fig. 5:**
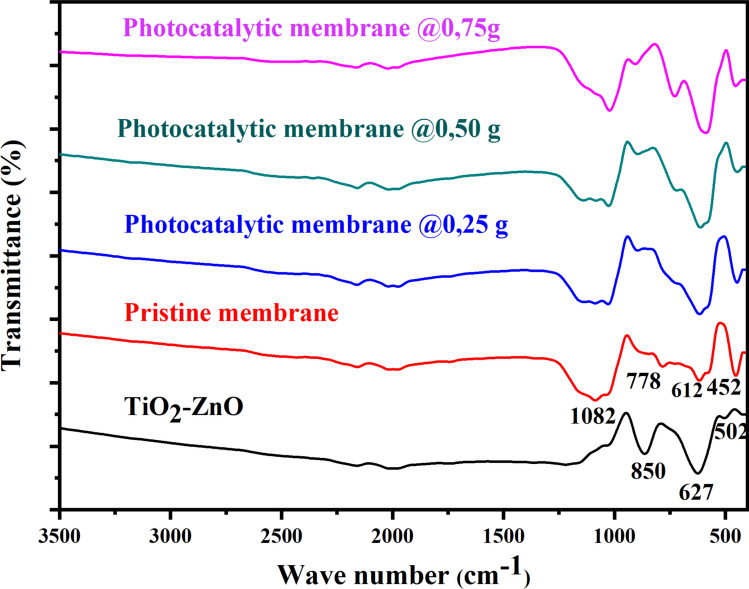
The FTIR spectra of TiO_2_–ZnO (black), pristine coal fly ash ceramic membrane (red), and functionalized ceramic membranes at different photocatalyst loading of 0.25 g (blue), 0.50 g (green), and 0.75 g (pink)

Figure 5 presents the infra-red spectra of TiO_2_–ZnO (black), pristine coal fly ash ceramic membrane (red), and functionalized ceramic membranes at different photocatalyst loading of 0.25 g, 0.50 g, and 0.75 g. The spectrum of TiO_2_–ZnO showed characteristic peaks around 850, 627, and 502 cm^−1^ which could be ascribed to the vibrational mode of Zn–O–Ti, vibration of Ti − O − Ti, and vibration of M − O linkages (where M represents Zn or Ti) respectively [44, 45]. The pristine ceramic membrane's FTIR spectrum displays asymmetric stretching vibration bands attributed to Si–O–Si around 1082 and 452 cm^–1^. Further, the pristine ceramic membranes also showed peaks at 612 and 778 cm^–1^, due to the stretching vibration of Al–O–Si [46]. The functionalized ceramic membranes’ spectra showed peaks consistent with pristine coal fly ash-based ceramic membrane and TiO_2_–ZnO nanoparticles, indicating the successful fabrication of photocatalytic ceramic membranes.

### Structural studies

**Fig. 6:**
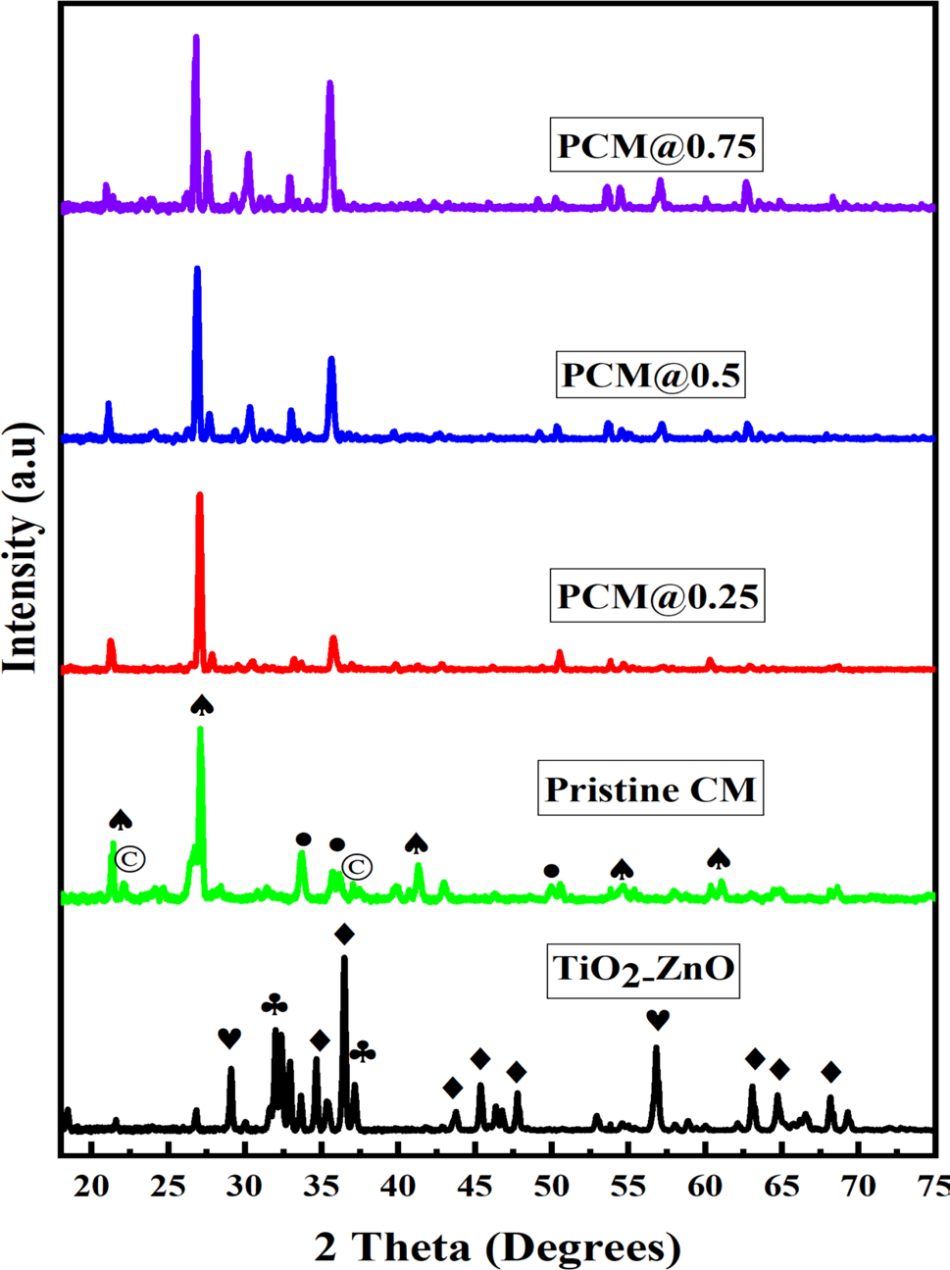
X-ray diffraction spectra of TiO_2_–ZnO (black), pristine coal fly ash ceramic membrane (green) and functionalized ceramic membranes at different photocatalyst loading of 0.25 g (red), 0.50 g (blue) and 0.75 g (purple). *(TiO*_*2*_* rutile (♥), ZnTiO*_*3*_* (♦), Zincite ZnO (♣), mullite (●), cristobalite (©) and α-quartz (♠)*

The X-ray diffraction patterns of TiO_2_–ZnO (black), pristine coal fly ash ceramic membrane (green), and photocatalytic ceramic membranes at different photocatalyst loading of 0.25 g (red), 0.50 g (blue), and 0.75 g (purple) are presented in Fig. 6. The diffraction patterns of the TiO_2_–ZnO heterojunction system confirmed the presence of rutile TiO_2_, hexagonal ZnTiO_3,_ and Zincite ZnO. The peaks for hexagonal ZnTiO_3_ appeared at 25, 34, 36, 43, 50, 54, 58, 63, and 64° and are in good agreement with JCPDS No. 26–1500 [47]. The characteristic peaks of rutile TiO_2_ were observed at 2θ = 27, 36, and 54° (JCPDS Card No. 21-1276) [48], while the characteristic peaks of Zincite ZnO were observed at 2θ = 33, 36, and 38° (JCPDS No. 65–3411) [49]. The sharp peaks confirm that the heterojunction photocatalyst contains crystalline components. The XRD patterns of the pristine coal fly ash-based ceramic membrane is composed of three major crystalline phases which are α-quartz (SiO_2_), cristobalite (tetragonal SiO_2_), and mullite (3Al_2_O_3_·2SiO_2_). The peaks for α-quartz (SiO_2_) are observed at 2θ values of 21.85, 26.85, 39.6, 49.5, 54.5, and 60.8°. The peaks due to the mullite and cristobalite were observed at 2θ values of (25.9, 33.7, 37.4, and 52.5°) and (22 and 36°) respectively. The presence of quartz and mullite phases confirmed the high thermal stability of the fabricated ceramic membrane. All the TiO_2_–ZnO peaks and those of pristine coal fly ash-based ceramic membranes were evident in the as-synthesized photocatalytic ceramic membranes demonstrating the success in functionalizing coal fly ash-based ceramic membranes with TiO_2_–ZnO photocatalyst. The crystallite size of the functionalized ceramic membranes estimated from the Scherrer equation (Eq. 10) was higher than that of the pristine ceramic membrane.

10 $$D \left( {Crystallite\, size \left( {nm} \right)} \right) = \frac{K\lambda }{{\beta Cos\theta }}$$

where K is the Scherrer’s constant (Shape factor) (0.94), λ is the Wavelength of X-rays (Å), β = F. W. H. M (Full Width at Half Maxima in degrees), θ = Peak position in XRD graph (that is 2θ in degrees).

### Thermal analysis

**Fig. 7:**
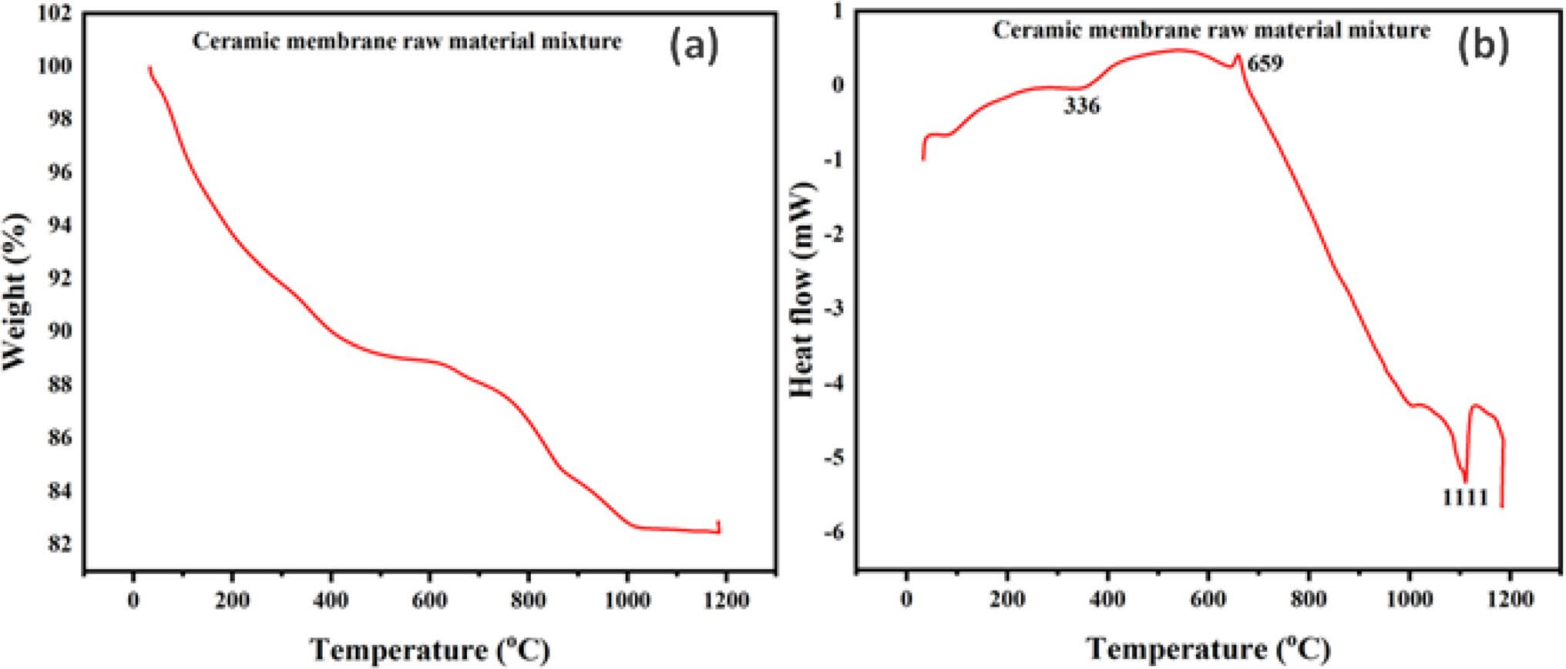
(**a**) Thermogravimetric (TG) and (**b**) Differential scanning calorimetry (DSC) graphs of ceramic membrane raw material mixture

The thermal stability properties of the materials were studied using Thermogravimetric analysis (TGA) and differential scanning calorimetry (DSC), and the results are shown in Fig. 7. The precursor material's TGA graph (Fig. 7) reveals a significant weight loss in two phases, with a total weight loss of almost 18%. The first loss observed below 450 °C was due to the removal of the adsorbed water and the decomposition of volatile organic matter, dextrin (pore former) and sodium carboxyl methyl cellulose (binder). This process was accompanied by a weak endothermic peak noted around 336 °C in the DSC thermogram (Fig. 7b). The increase in temperature from 450 to 1000 °C resulted in a weight loss of around 7%. A weak exothermic peak noted at 659 °C in the DSC curve is likely attributed to the incineration of unburnt carbon and mineral degradation. When the temperature was further increased to 1200 °C, a narrow endothermic peak appeared in the DSC curve, which could be due to the phase transformation of quartz [50, 51].

### Morphological studies

**Fig. 8:**
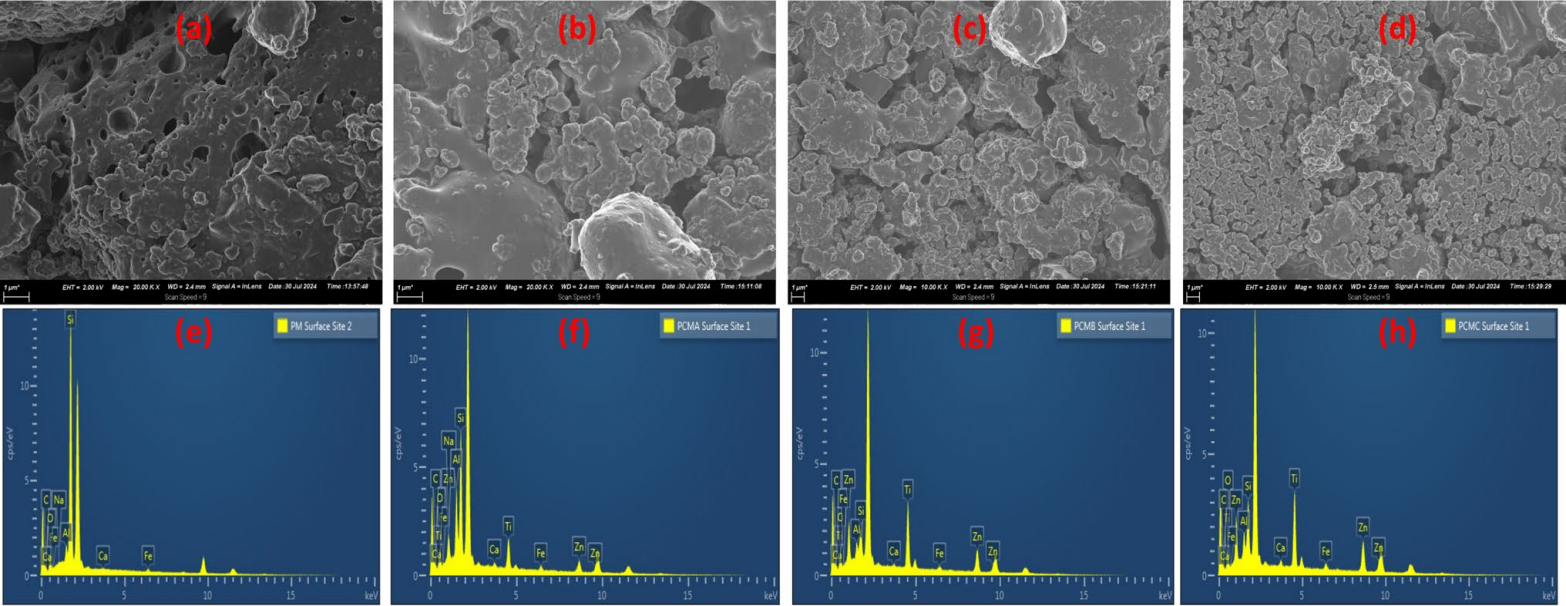
SEM surface micrographs of (**a**) pristine coal fly ash ceramic membrane and functionalized ceramic membranes at different photocatalyst loading of (**b**) 0.25 g, (**c**) 0.50 g, and (**d**) 0.75 g; and the EDX spectra of (**e**) pristine coal fly ash ceramic membrane and functionalized ceramic membranes at different photocatalyst loading of (**f**) 0.25 g, (**g**) 0.50 g, and (**h**) 0.75 g

The morphological changes of the synthesized membranes with varying loads of TiO_2_–ZnO and their respective elemental composition are presented in Fig. 8a–h. The micrograph of the pristine ceramic membranes reveals a distinct and well-distributed pore structure. In contrast, upon functionalization, there is a noticeable reduction in pore size and distribution as the TiO_2_–ZnO load was gradually increased from 0.25 to 0.75 g. This pattern, thus, indicates that a larger surface coverage with densely packed spherical particles was obtained due to the increase of TiO_2_–ZnO on the surface, thereby decreasing the overall pore density. The transition in surface morphology from the pristine ceramic membranes' darker appearance to the lighter appearance of the functionalized membranes further corroborates the successful functionalization process (Fig. 8a–d. The observed colour change indicates the effective deposition of the TiO_2_–ZnO particles on the membrane surface. Furthermore, the surface micrographs of the base of both pristine and functionalized ceramic membranes show uniformity in colour and pore distribution, indicating that the functionalization process primarily affected the top surface morphology. The EDX spectra of the fabricated membranes are displayed in Figs. 8e–h. Si, Al, Fe, and Ca are the major elements found in the pristine coal fly ash-based ceramic membranes. The additional Ti and Zn that appeared on the surface of functionalized coal fly ash-based ceramic membranes are due to the functionalization. This further confirms the successful incorporation of TiO_2_–ZnO heterojunction incorporation into the ceramic matrix.

### Photocatalytic activity of the functionalized ceramic membranes

#### Effect of solution pH

**Fig. 9:**
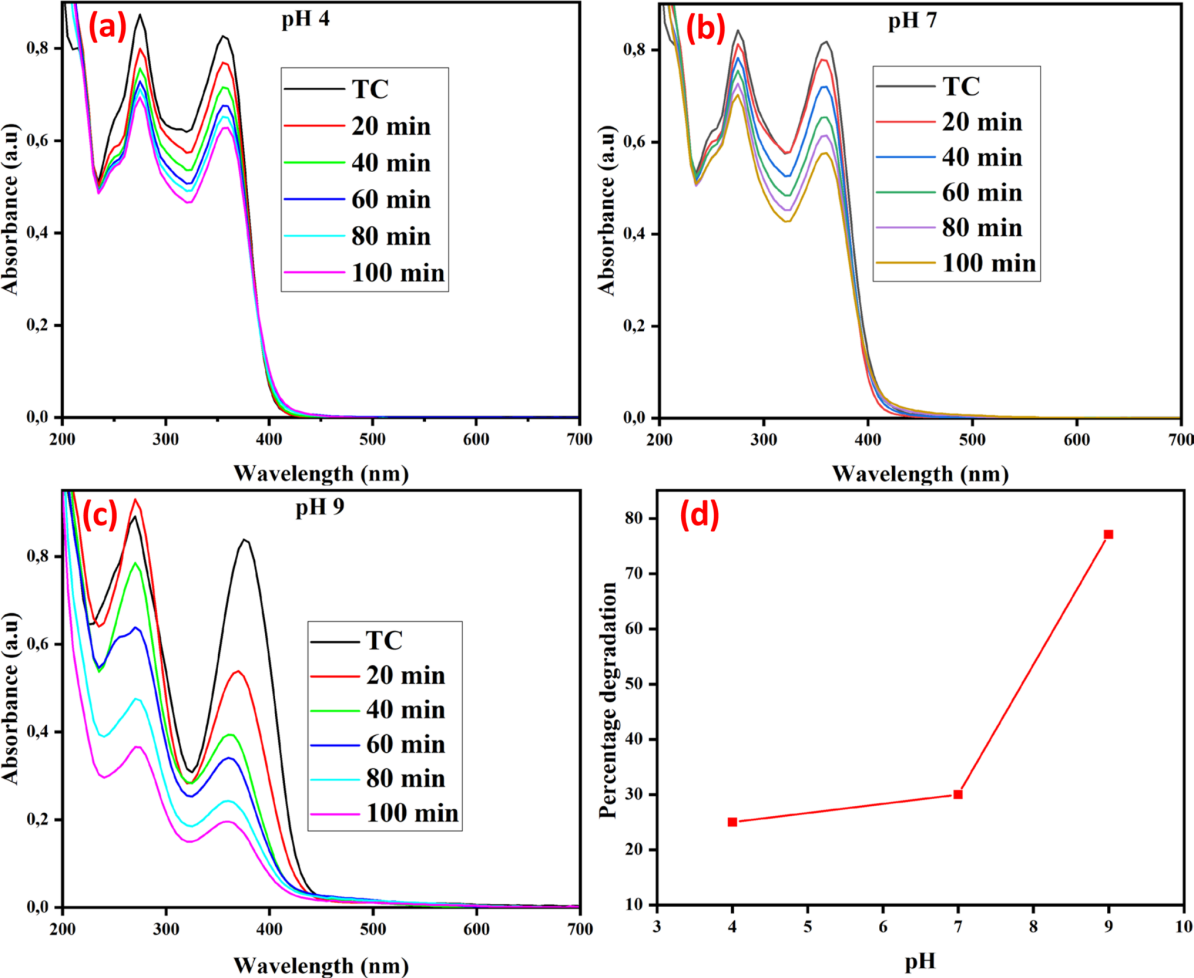
Photocatalytic degradation profile of Tetracycline by TiO_2_–ZnO functionalized coal fly ash-based ceramic membranes at different pH (**a**) 4, (**b**) 7, and (**c**) 9. (**d**) Percentage degradation of TC under visible light (TC initial concentration: 25 mg/L)

Solution pH is an important factor in photocatalytic degradation study. This is because it influences the surface electrical charge that is characteristic of the catalyst and dictates the ionization state of the catalyst's surface [52]. Figure 9 shows the effect of pH on the degradation of tetracycline using a TiO_2_–ZnO functionalized coal fly ash-based ceramic membrane. A model solution of tetracycline with a concentration of 25 mg/L and a coal fly ash-based ceramic membrane functionalized with 0.5 g of TiO_2_–ZnO heterojunction were used. The study was conducted over 100 min. The initial 20 min was for a steady-state adsorption–desorption equilibrium to be established, while the remaining 80 min were used to facilitate the photocatalytic degradation process. It is evident from the results that pH significantly affects the degradation of TC by the functionalized ceramic membranes as pH of 4,7 and 9 resulted in degradation efficiency of 25, 29 and 77.6% respectively (Fig. 9d). The results corroborate well with previously reported studies that used black TiO_2_–ZnO [20] and nanosized TiO_2_ [53]. The pH of the solution changes the surface charges of tetracycline and influences the adsorption process, so an increase in adsorption and photocatalytic activity at pH 9 could be attributed to the electrostatic interaction between the charges of TC species and the functionalized ceramic membrane [54]. This could also be attributed to tetracycline deprotonation, which increases its adsorption onto the photocatalytic surface. Furthermore, the increased availability of hydroxyl radicals at alkaline pH enhances the degradation process [55]. Tetracycline is an amphoteric molecule with pKa values of 3.32, 7.78, and 9.58, and it exists in three forms depending on the pH. At pH < 3.32, it is primarily cationic, between pH 3.32 and 7.78 it is zwitterionic and above pH 7.78, it becomes anionic [56]. Tetracycline molecules with negative charge generate reactive species such as OH^•^ due to high electrical density on the ring system, thereby enhancing the degradation efficiency of tetracycline. The optimization of pH in real wastewater treatment applications is important since changes in pH can affect both the ionization state of pollutants and the surface charge of the photocatalyst, which may lead to reduced adsorption and, consequently, decreased degradation efficiency*.* The pH of 9 was therefore chosen for the subsequent evaluation of the photocatalytic degradation of TC in this study.

#### Effect of tetracycline concentration

**Fig. 10:**
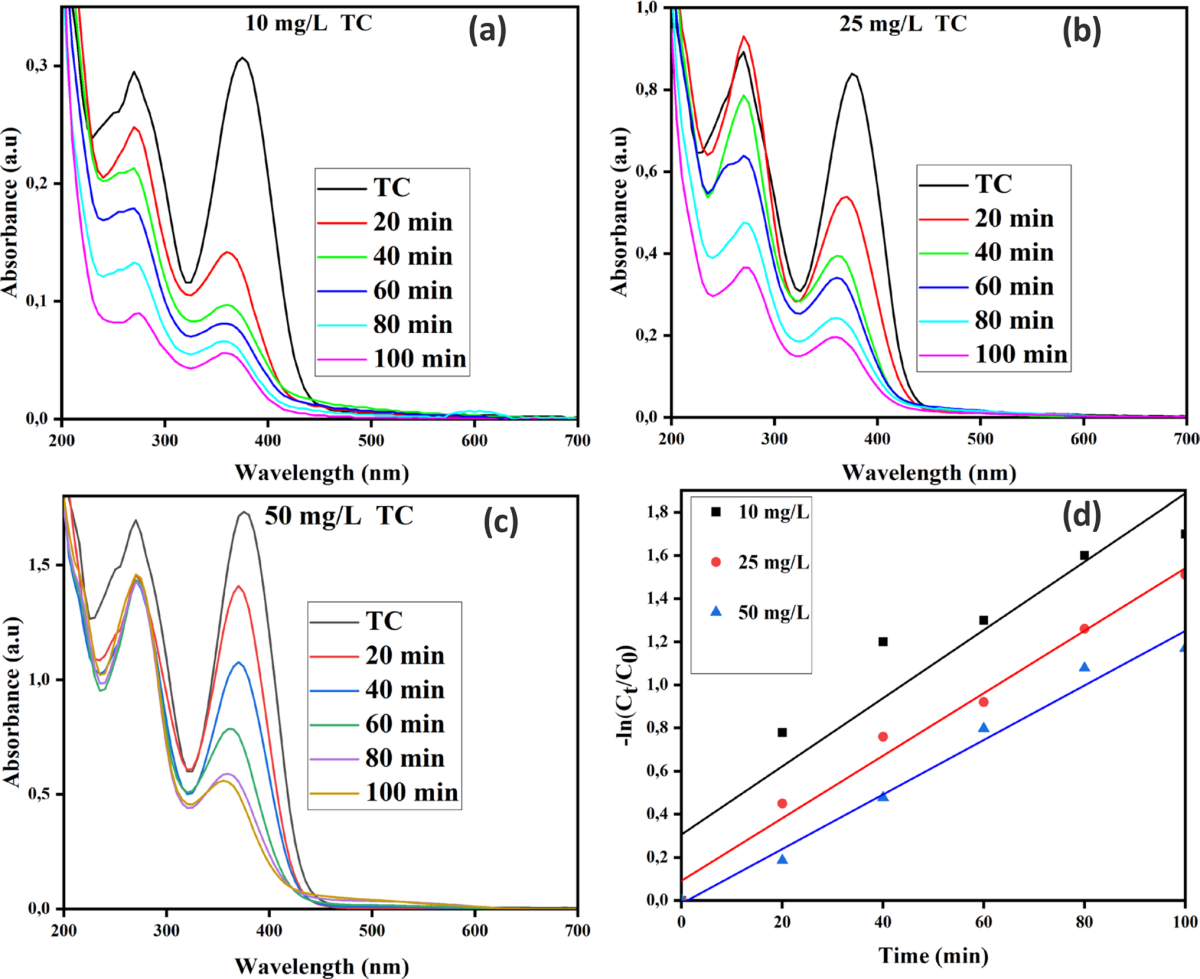
Effect of Tetracycline concentration at (**a**) 10 mg/L, (**b**) 25 mg/L, and (**c**) 50 mg/L on the photocatalytic degradation of Tetracycline by TiO_2_–ZnO functionalized coal fly ash-based ceramic membranes and (**d**) the kinetic plots for the effect of antibiotic concentration on the photocatalytic degradation of Tetracycline

Figure 10 shows the effect of initial TC concentration on the degradation efficiency of the process. TiO_2_–ZnO functionalized coal fly ash-based ceramic membranes demonstrated effective TC degradation across the entire concentration range examined. However, degradation efficiencies decreased as TC concentration increased reaching 82%, 77.6%, and 68.8% at 10, 25, and 50 mg/L, respectively. The reaction rate constants also decreased as the initial concentration of tetracycline increased, and at 10, 25, and 50 mg/L, they were 0.016, 0.014, and 0.013 min^−1^, respectively. The observed decline in degradation efficiency and reaction rates at higher concentrations may be attributed to several factors. At high tetracycline (TC) concentrations, TC ions dominate the photocatalyst's active sites, which hinders photons from reaching the functionalized ceramic membrane surface. This reduces the photocatalytic activity due to a decrease in the amount of hydroxyl radicals produced. Since the photocatalytic ceramic membrane and the tetracycline (TC) model wastewater exists in different phases, the rate of photocatalytic degradation is significantly influenced by mass transfer and diffusion between these phases. Therefore, the system also changes from a kinetic control domain, where reaction rates are driven by intrinsic kinetics at low concentrations, to a mass transfer-limited regime at high concentrations, where the reaction rate is limited by the efficiency of reactant delivery to the reaction site [57]. As the concentration of TC increases, mass transfer limitations may become more pronounced, potentially reducing the availability of TC at the active surface of the photocatalytic membrane [58]. High TC concentrations can create thicker concentration gradients near the membrane, which can limit the effective mass transfer and hinder their interaction with the photocatalyst. The effectiveness of the degradation process may be impacted by the intermediates that tetracycline molecules may generate during photocatalytic activities. These intermediates may compete with the catalyst's surface for photocatalytic and adsorption sites [59]. These results are similar to those reported by Pourshaban-Mazandarani and Nasiri [60] and Deng et al.[61]**.**

#### Effect of photocatalyst loading

The effect of varied photocatalyst loadings on the breakdown of 25 mg/L tetracycline was investigated using a functionalized coal fly ash-based ceramic membrane. Figure 12a depicts an increase in the rate of tetracycline degradation as the photocatalyst loading increased from 0.25 to 0.75 g. The enhanced rate of photocatalytic degradation was caused by a rise in the number of surface-active sites, which resulted in the increased creation of photoactive species [62]. However, the increase in photocatalyst loading resulted in reduced adsorption of tetracycline during the initial 20-min adsorption–desorption equilibrium. This reduction may be attributed to the saturation of available adsorption sites on the photocatalyst or possible steric hindrance that limits the access of tetracycline molecules to the ceramic membrane surface [63]. Additionally, a higher photocatalyst concentration may lead to more competitive interactions, hindering effective adsorption of the tetracycline onto the catalyst surface [64]. Similar results were reported during the photocatalytic degradation of tetracycline hydrochloride over AgBr nanoparticles decorated GO/Bi_2_WO_6_ photocatalyst [65].

#### Synergistic effect of adsorption and photocatalysis

**Fig. 11:**
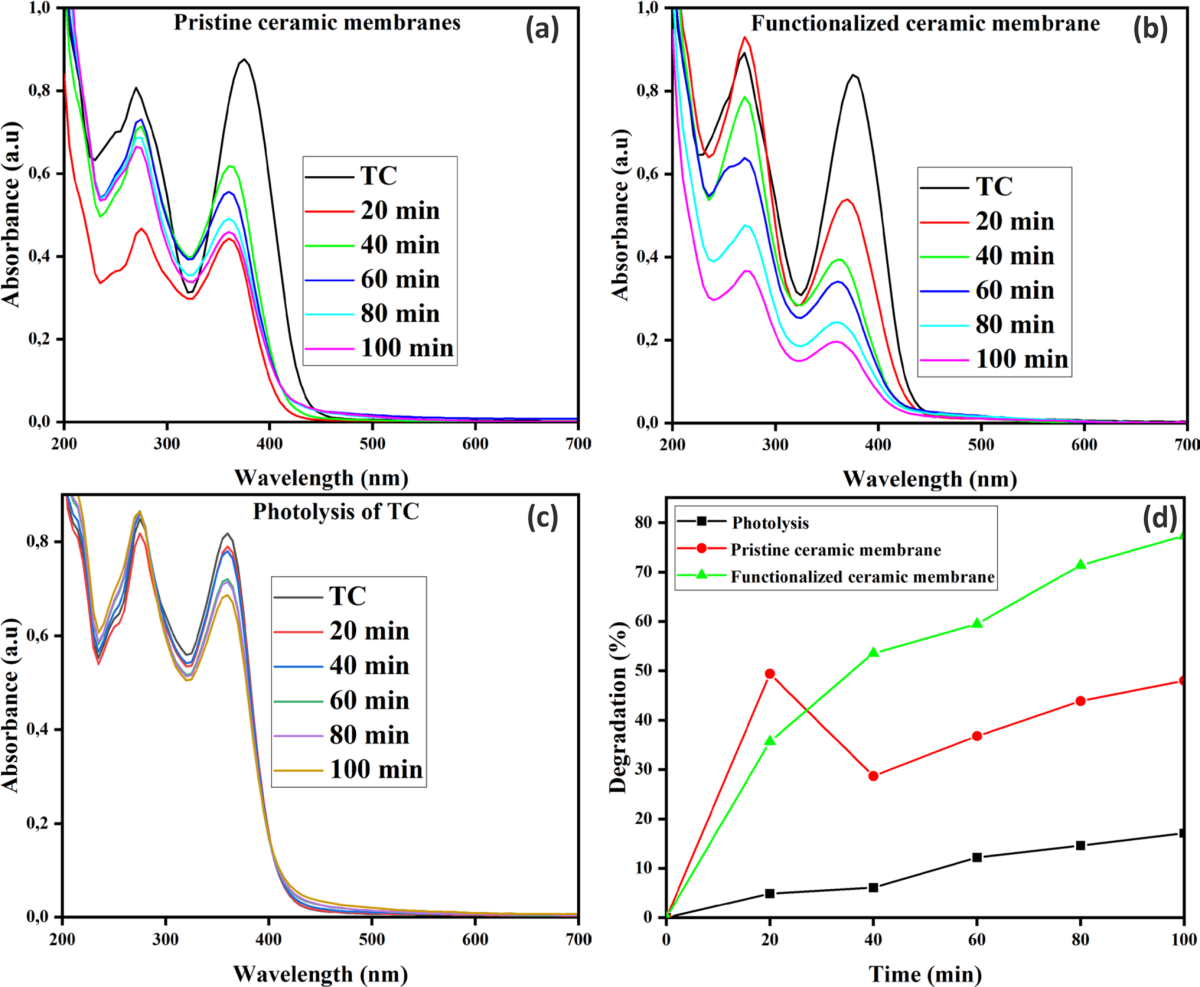
Degradation profile of (**a**) Pristine coal fly ash based ceramic membrane, (**b**) TiO_2_–ZnO functionalized coal fly ash based ceramic membrane, (**c**) Photolysis of Tetracycline and (**d**) Percentage degradation of TC under visible light

Figures 11a and b show tetracycline removal efficiency by the pristine coal fly ash-based ceramic membrane and TiO_2_–ZnO functionalized ceramic membrane. During the first 20 min of the adsorption studies, the pristine coal fly ash-based ceramic membrane showed a higher tetracycline removal efficiency of 49% compared to the functionalized ceramic membrane, which achieved only 35%. This suggests that the pristine membrane may have a greater affinity for tetracycline in the initial stages, possibly due to its surface properties or pore structure that favors adsorption [66, 67]. The Si–O-Si and Al–O–Si groups found in pristine ceramic membranes can generate a surface with polar characteristics. Since tetracycline is a polar organic molecule, it can interact more favorably with these functional groups, resulting in enhanced adsorption through dipole–dipole interactions [68]. After exposure to visible light, the functionalized ceramic membrane exhibited a marked improvement in tetracycline removal efficiency, reaching 77.6% after 100 min of irradiation. This indicates that the TiO_2_–ZnO functionalization significantly enhances the photocatalytic activity of the ceramic membrane, facilitating the degradation of tetracycline under visible light exposure. In contrast, the pristine coal fly ash ceramic membrane's degradation efficiency declined after the initial adsorption phase, with its removal efficiency after 100 min being lower than the initial 49%. This suggests a paucity of photocatalytic activity and demonstrates that the pristine membrane is predominantly functional through adsorption. The subsequent increase in tetracycline concentration following adsorption reflects a desorption process in which tetracycline is released back into the solution rather than mineralized or further degraded. The results corroborate the hypothesis that ceramic membranes do not mineralize pollutants; rather, pollutants appear to shift from one phase (adsorbed) to another (dissolved). The effect of photolysis was also evaluated in Fig. 11 c. The results showed an insignificant impact of direct photolysis of tetracycline at pH of 9 as evidenced by degradation efficiency of less than 12%.

#### Reusability of the functionalized ceramic membrane

**Fig. 12:**
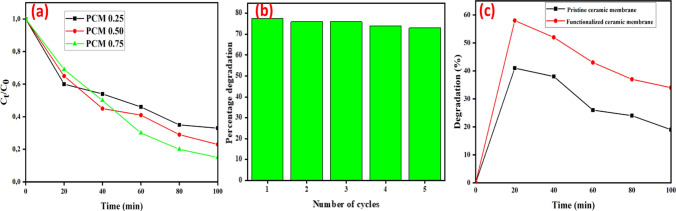
**a** Rate of TC removal with varying TiO_2_–ZnO loadings on coal fly ceramic membranes, **b** Reusability of TiO_2_–ZnO functionalized coal fly ash-based ceramic membrane for tetracycline degradation, **c** Tetracycline removal efficiency by pristine coal fly ash-based ceramic membrane and TiO_2_–ZnO (0.5 g) functionalized coal fly ash-based ceramic membrane through adsorption and filtration (TC concentration 25 mg/L, solution pH = 9, time 100 min)

The reusability of the membrane was assessed by 5-cycle runs and Fig. 12b shows the results obtained from the functionalized coal fly ash-based ceramic membrane using 25 mg/L TC concentration. After each cycle, the functionalized ceramic membrane was washed with water and then dried at 150 °C for 10 h. The membrane showed high stability and reusability by achieving 73% degradation efficiency after the 5th photocatalytic cycle. This effective reusability can be attributed to the limited loss of the photocatalyst since it is not directly recovered from the solution [69].

### Synergistic effect of adsorption and filtration performance

In Fig. 12c, the tetracycline removal efficiency by pristine coal fly ash-based ceramic membrane and TiO_2_–ZnO functionalized coal fly ash-based ceramic membrane (through adsorption and filtration) are shown. During the first 20 min, both membranes showed excellent removal effectiveness, with the functionalized ceramic membrane outperforming the pure coal fly ash-based ceramic membrane. This was due to the presence of nano-sized pores, which provide a larger surface area and more active sites for filtration, allowing for more effective contaminants removal, whereas the pristine coal fly ash-based membrane has pores in the micrometer range, resulting in fewer and less effective filtration sites, causing a lower initial removal efficiency compared to the functionalized ceramic membrane. The decrease in removal efficiency after 20 min by both membranes can be attributed to the saturation of the adsorption sites on the membrane surfaces and the passage of the trapped elements in the pores into the permeate [70, 71]. The deposition of particles on the membrane surface and the pores leads to the fouling of the membrane [72].

#### Photocatalytic mechanism

**Fig. 13:**
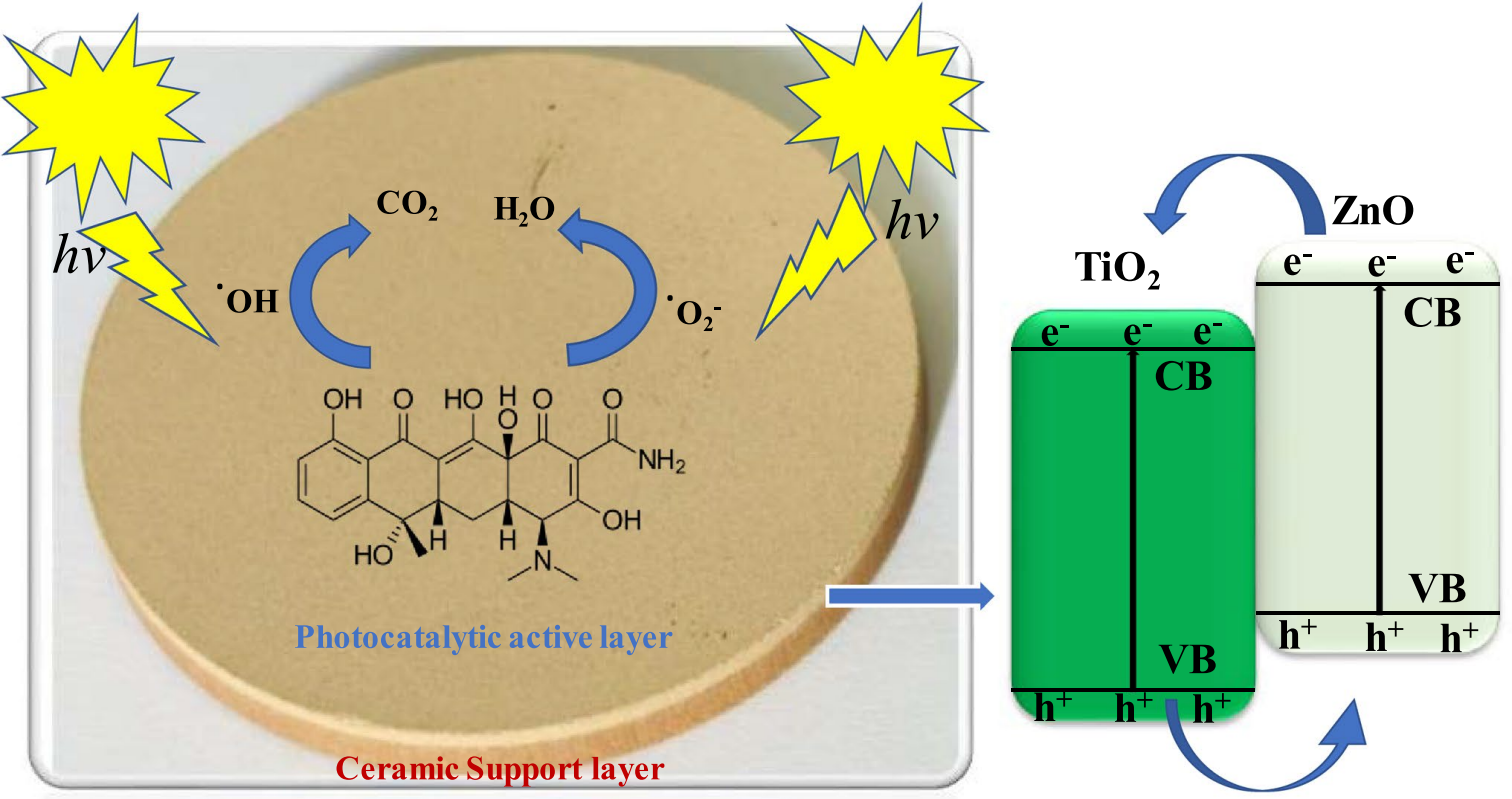
Photocatalytic degradation mechanism of Tetracycline by TiO_2_–ZnO functionalized coal fly ash-based ceramic membrane

A photocatalytic degradation mechanism of the tetracycline by TiO_2_–ZnO functionalized ceramic membrane has been proposed and presented in Fig. 13. Firstly, the tetracycline molecules are attracted to and adhere to the surface of the photocatalytic membrane. Upon exposure to visible light, the photocatalysts TiO_2_ and ZnO absorb some photons, generating electrons and holes. The energy from the light excites electrons from the valence band to the conduction band, and the generated electrons react with dissolved oxygen to form superoxide anion radicals. Simultaneously, holes can oxidize water or hydroxyl ions to form hydroxyl radicals. These superoxide anions and hydroxyl radicals then attack the adsorbed tetracycline molecules, breaking down their structures into less harmful compounds. The intermediate products are further oxidized, ultimately leading to the complete mineralization of tetracycline into carbon dioxide and water (H₂O). After the degradation process, some of the remaining by-products may still be adsorbed on the membrane surface after filtration [73, 74].

## Conclusion

A sustainable and cost-effective ceramic membrane functionalized with TiO_2_–ZnO heterojunction photocatalyst was fabricated using co-pressing and co-sintering methods. Coal fly ash and natural sand were used as the main starting materials. The functionalized coal fly ash-based ceramic membrane exhibited nanoscale pore sizes on its photocatalytic surface and excellent thermal, chemical, and mechanical stability. The functionalized ceramic membrane showed 77.6% removal efficiency by photocatalysis and 57% removal efficiency by filtration for model wastewater containing 25 mg/L concentration of tetracycline. In filtration studies, tetracycline was simply adsorbed and filtered without the degradation complicating desorption from surface-active areas. This requires special desorption techniques to regenerate the membrane, which is expensive and involves high re-sintering temperatures to break down the retained tetracycline. This has the potential to cause environmental problems. Therefore, the fabricated functionalized ceramic membrane could be used in a hybrid photocatalysis and filtration reactor system to optimize its synergistic photocatalytic, adsorption, and filtration properties. Future studies could also investigate the photocatalytic degradation of a broader range of emerging contaminants, including pharmaceuticals (e.g., antibiotics other than TC), personal care products, organic dyes, and endocrine-disrupting compounds. This would provide a more comprehensive understanding of the efficiency of this hybrid wastewater treatment.

## Data Availability

The datasets generated during and/or analyzed during the current study are available from the corresponding author upon reasonable request.
